# CD63^hi^ macrophages define a conserved terminal inflammatory state driving lung immunopathology in severe COVID-19

**DOI:** 10.3389/fcimb.2026.1874567

**Published:** 2026-08-21

**Authors:** Yaoxi Tan, Jie Yang, Mingming Rong, Zhou Cheng, Kangkang Ji

**Affiliations:** 1 Department of Infectious Diseases, Affiliated Hospital of Jiangnan University, Wuxi, Jiangsu, China; 2 Department of Respiratory and Critical Care Medicine, Binhai County People’s Hospital, Binhai Clinical College, Yangzhou University Medical College, Yancheng, Jiangsu, China; 3 College of Biomedicine and Health, Huazhong Agricultural University, Wuhan, Hubei, China

**Keywords:** CD63, COVID-19, *ELAVL1*, inflammatory, monocyte-derived macrophages

## Abstract

**Background:**

Severe COVID-19 is characterized by profound lung immune dysregulation, yet the pathogenic states and regulatory mechanisms of monocyte-derived macrophages (MDMs) remain poorly defined due to substantial phenotypic heterogeneity across studies.

**Methodology:**

We integrated multiple single-cell RNA sequencing datasets from COVID-19 lung tissues and bronchoalveolar lavage fluid (BALF) samples to systematically characterize macrophage heterogeneity and differentiation dynamics across anatomical compartments. Trajectory inference, transcription factor activity analysis, and m^6^A-associated regulatory profiling were performed to delineate molecular programs underlying macrophage state transitions. Cross-dataset validation analyses were conducted to assess the conservation and clinical relevance of identified macrophage states. Functional roles of candidate regulators were further investigated through *ELAVL1* silencing in monocyte-derived macrophages *in vitro*, while the pathogenic potential of CD63^hi^ macrophages was evaluated using adoptive transfer experiments in an acute lung injury mouse model.

**Results:**

We define a conserved CD63^hi^ terminal inflammatory macrophage state markedly expanded in severe COVID-19 lungs. CD63^hi^ macrophages exhibited enhanced inflammatory cytokine expression together with elevated glycolysis- and hypoxia-associated programs, representing a terminal inflammatory differentiation state along the monocyte-to-MDM trajectory. This population was reproducibly detected across independent datasets and preferentially enriched in lung tissues, with strong associations with disease severity and adverse clinical outcomes. Transcriptional analysis identified *JUND* activity as progressively increased during macrophage differentiation toward the CD63^hi^ state. In parallel, *ELAVL1* was associated with inflammatory and metabolic reprogramming programs. *ELAVL1* silencing suppressed pro-inflammatory cytokine expression, attenuated glycolytic and hypoxia-related pathways, and shifted macrophages toward an immune-regulatory phenotype accompanied by reduced *CD63* expression. Functionally, adoptive transfer of CD63^hi^ macrophages exacerbated lung inflammation *in vivo*, resulting in increased cytokine production and neutrophil infiltration.

**Conclusions:**

In conclusion, our study defines a conserved CD63^hi^ macrophage inflammatory state in severe COVID-19 and reveals its association with metabolic reprogramming, lung immunopathology, and disease severity. *ELAVL1* emerges as an important RNA regulatory factor associated with inflammatory macrophage remodeling, highlighting the potential of targeting macrophage regulatory networks as a strategy for modulating pathological inflammation in severe viral infection.

## Introduction

Coronavirus disease 2019 (COVID-19) is characterized by profound immune dysregulation in the lung, and excessive inflammation is a major driver of disease severity and mortality (Melms et al., 2021; Ren et al., 2021; Stephenson et al., 2021). Although antiviral responses are essential for viral clearance, uncontrolled immune activation-particularly within the myeloid compartment-contributes to tissue damage and respiratory failure. Recent studies have reported extensive remodeling of the pulmonary immune microenvironment in COVID-19 (Melms et al., 2021). However, the cellular sources and regulatory mechanisms driving pathogenic inflammation remain incompletely defined and, in some cases, inconsistently described across studies.

Macrophages are central components of the lung immune landscape and play critical roles in coordinating host defense, inflammation, and tissue repair (Aegerter et al., 2022; Lazarov et al., 2023; Lin et al., 2026; Rodriguez-Morales and Franklin, 2023; Yang et al., 2023). In COVID-19, monocyte-derived macrophages (MDMs) are markedly expanded and have been implicated as major producers of inflammatory cytokines (Melms et al., 2021). However, accumulating evidence suggests that macrophage responses are highly heterogeneous (Mitsui and Satoh, 2025; Russell et al., 2025; Zhang et al., 2026), and the specific subsets responsible for protective versus pathogenic functions remain poorly resolved. Notably, existing markers are insufficient to distinguish functionally distinct inflammatory macrophage states, limiting both mechanistic understanding and the development of targeted therapeutic strategies. Moreover, it remains unclear whether these inflammatory states represent stable lineages or dynamic transitions shaped by the inflammatory microenvironment.

Emerging evidence indicates that macrophage activation is tightly coupled to metabolic and transcriptional reprogramming (Ge et al., 2025; Gong et al., 2025; Jin et al., 2025; Wang et al., 2025). Inflammatory macrophages often exhibit enhanced glycolysis and hypoxia-associated signaling, supporting rapid cytokine production and adaptation to inflamed tissues (Kelly and O'Neill, 2015; Mirchandani et al., 2025; Ting, 2024). While several transcription factors have been implicated in macrophage activation (Dominguez-Lopez et al., 2025; Liu et al., 2025; Mussbacher et al., 2023), the regulatory networks governing macrophage state transitions in COVID-19 remain poorly defined. In addition, post-transcriptional mechanisms have recently emerged as critical regulators of immune cell function. In particular, m^6^A-related regulators that control mRNA stability and translation may play pivotal roles in shaping macrophage responses (Chen et al., 2025; Jiao et al., 2026; Wang et al., 2026), yet their contribution to inflammatory macrophage states in COVID-19 has not been systematically investigated.

In this study, we sought to resolve macrophage heterogeneity and define the regulatory framework underlying pathogenic inflammation in COVID-19 through integrative single-cell transcriptomic analyses combined with experimental validation. We identified a distinct CD63^hi^ inflammatory macrophage population that is selectively enriched in COVID-19 lungs and occupies a terminal position along the monocyte-to-macrophage differentiation trajectory. These CD63^hi^ macrophages exhibit robust pro-inflammatory and metabolically reprogrammed features and are consistently associated with disease severity across independent datasets. Mechanistically, we identify a regulatory framework involving the RNA-binding protein ELAVL1 and the transcription factor JUND, both of which are associated with the inflammatory remodeling of CD63^hi^ macrophages. *ELAVL1* contributes to the maintenance of inflammatory and metabolic programs associated with CD63^hi^ macrophages. Functionally, CD63^hi^ macrophages exacerbate lung inflammation *in vivo*, highlighting their direct contribution to disease pathology. Collectively, our findings define a previously uncharacterized CD63^hi^ inflammatory macrophage state and establish a mechanistic link between macrophage differentiation, regulatory programming, and immunopathology in COVID-19.

## Materials and methods

### Data sources

Publicly available single-cell and single-nucleus RNA sequencing (scRNA-seq/snRNA-seq) datasets of COVID-19 patients were obtained from the Gene Expression Omnibus (GEO) database. To investigate macrophage populations across different tissues and disease conditions, three datasets were included in this study: GSE171524, GSE171668, and GSE158055. The dataset GSE171524 consists of single-nucleus transcriptomic profiles generated from postmortem lung tissues of COVID-19 decedents and control individuals. The dataset GSE171668 includes single-cell RNA sequencing data from multiple organs, including lung, heart, liver, and kidney tissues collected from COVID-19 autopsy donors. The dataset GSE158055 contains single-cell transcriptomic data from peripheral blood, bronchoalveolar lavage fluid (BALF), and additional respiratory samples obtained from healthy controls, COVID-19 patients with varying disease severities, and convalescent individuals. These datasets encompass multiple tissue types and clinical conditions and were jointly analyzed to characterize mononuclear phagocyte populations across compartments.

### Single-cell data processing and data analysis

Raw count matrices were processed using the Seurat package (v4.3.0) (Hao et al., 2021) in R (v4.2.1) (https://www.r-project.org/). Cells with low-quality metrics were excluded based on the following criteria: mitochondrial gene proportion > 20%, total Unique Molecular Identifier (UMI) counts (nCount_RNA) < 100 or > 15,000, and detected gene numbers (nFeature_RNA) < 200 or > 10,000. Gene expression data were normalized using the NormalizeData function. Highly variable genes were identified using the FindVariableFeatures function with mean expression values between 0.0125 and 3 and variance > 0.5. The data were then scaled using the ScaleData function. Principal component analysis (PCA) was performed on the scaled data, and the top principal components capturing the majority of variance were used for downstream analyses. To correct for batch effects across samples, the Harmony algorithm was applied to the PCA embeddings using donor identity as the batch variable. The corrected embeddings were used for dimensionality reduction with Uniform Manifold Approximation and Projection (UMAP). A shared nearest-neighbor (SNN) graph was constructed using the FindNeighbors function, followed by unsupervised clustering using the FindClusters function with a resolution parameter of 1.0. Cell-type annotation was performed using a combination of automated and manual approaches. Automated annotation was conducted with the SingleR package (v2.0.0), and results were further refined based on canonical marker gene expression. Downstream analyses were performed on the processed data. Pseudotime trajectory analysis was conducted using the Monocle2 package (Trapnell et al., 2014), and gene regulatory network inference was performed using SCENIC (Aibar et al., 2017).

### Detection of differentially expressed genes and functional enrichment analysis

Differentially expressed genes (DEGs) were identified using the Seurat package (Hao et al., 2021). For cluster-level comparisons, the FindAllMarkers function was applied with the Wilcoxon rank-sum test. For comparisons between experimental conditions, the FindMarkers function was used. In all analyses, genes expressed in at least 25% of cells, with a log2 fold change greater than 0.5 and a false discovery rate (FDR) below 0.05, were considered statistically significant. Functional enrichment analysis of DEGs was performed using the clusterProfiler package. Terms with a *P*-value < 0.05 and q-value < 0.05 were considered significantly enriched.

### m^6^A regulator related gene set

A curated set of 22 m^6^A regulators was analyzed, including methyltransferases (“writers”) and associated cofactors (*METTL3, METTL14, WTAP, RBM15, RBM15B, ZC3H13*), demethylases (“erasers”) (*FTO, ALKBH5*), and RNA-binding proteins (“readers”) (*YTHDF1, YTHDF2, YTHDF3, YTHDC1, YTHDC2, IGF2BP1, IGF2BP2, IGF2BP3, HNRNPC, HNRNPA2B1, FMR1, LRPPRC, ELAVL1, CBLL1*). Gene symbols were standardized according to HGNC nomenclature.

### Pathway signature scoring and regulator-pathway correlation analysis

Cell-level pathway activity scores were computed using the Seurat AddModuleScore function based on log-normalized expression data. Gene signatures included immune regulatory, inflammatory, hypoxia, glycolysis, and immunity hub-associated gene sets (Lin et al., 2026). Pathway scores were scaled within each cell lineage for downstream analyses. Associations between regulator expression and pathway activity scores were evaluated using Spearman correlation within each cell type. *P*-values were derived from the correlation tests, and multiple testing correction was performed using the Benjamini-Hochberg procedure to control the false discovery rate (FDR). Correlation coefficients and adjusted *P*-values were reported. Heatmaps were used to visualize the full set of correlations.

### m^6^A-related regulator-high and regulator-low contrast analysis in MDMs

Within MDMs populations (MDM_1, MDM_2, MDM_3, and MDM_4), cells were stratified into *ELAVL1*-high and *ELAVL1*-low groups based on the median expression of *ELAVL1*. Differentially expressed genes (DEGs) between the two groups were identified using the Wilcoxon rank-sum test. Genes with an absolute log2 fold change ≥ 0.25, expressed in at least 10% of cells in either group (min.pct ≥ 0.10), and a false discovery rate (FDR) < 0.05 were considered significant. Gene Ontology (GO) enrichment analysis of DEGs was performed using the clusterProfiler package (Wu et al., 2021). Terms with an adjusted FDR < 0.05 were considered significantly enriched.

### PBMC-derived macrophage differentiation and siRNA transfection

Peripheral blood mononuclear cells (PBMCs) were isolated from healthy donor blood by Ficoll density gradient centrifugation. Monocytes were obtained through adherence and cultured in RPMI 1640 medium supplemented with 10% fetal bovine serum (FBS) and macrophage colony-stimulating factor (M-CSF; CHAMOT Biotechnology, Cat# CM061-20MP) for 5–7 days to induce differentiation into macrophages. For gene silencing, macrophages were transfected with small interfering RNA (siRNA) targeting *ELAVL1* or a non-targeting control using Lipofectamine™ 2000 (Invitrogen) according to the manufacturer’s instructions. The siRNA duplexes were commercially synthesized (Sangon Biotech, Shanghai). After 6 h of transfection, the medium was replaced with fresh complete medium, and cells were allowed to recover overnight prior to stimulation. For inflammatory activation, macrophages were stimulated with lipopolysaccharide (LPS; typically 100 ng/mL) alone or in combination with tumor necrosis factor-α (TNF-α; 10 ng/mL) for the indicated durations to mimic inflammatory conditions. Following stimulation, cells were harvested for downstream RNA and protein analyses.

### RNA immunoprecipitation-qPCR assay

To assess the potential RNA-binding activity of ELAVL1 in inflammatory macrophages, RNA immunoprecipitation followed by quantitative PCR (RIP-qPCR) was performed using PBMC-derived macrophages. Briefly, macrophages differentiated as described above were stimulated with LPS (100 ng/mL) for the indicated duration to induce an inflammatory state prior to RIP analysis. Cells were harvested and lysed using RIP lysis buffer (Thermo Scientific™, Cat# 87788) supplemented with RNase inhibitors (Thermo Scientific™, Cat# EO0381) and protease inhibitors (Thermo Scientific™, Cat# 78410). Cell lysates were incubated with magnetic beads conjugated with an anti-ELAVL1 antibody (Abcam, Cat# ab28660) or control IgG antibody (Abcam, Cat# ab172730) overnight at 4 °C with gentle rotation.

Following immunoprecipitation, beads (Thermo Scientific™, Cat# 10003D) were extensively washed to remove non-specific interactions, and RNA-protein complexes were subsequently extracted using phenol or chloroform-based RNA isolation. The purified RNA from input and immunoprecipitated samples was reverse-transcribed into cDNA, and the enrichment of candidate transcripts was quantified by qRT-PCR. Candidate genes were selected based on their involvement in inflammatory activation, glycolytic metabolism, and hypoxia-associated programs enriched in CD63^hi^ macrophages, including *TNF*, *IL1B*, *CXCL8*, *PFKL*, *PGK1*, *LDHA*, and *HIF1A*.

### Adoptive transfer of macrophages in an LPS-induced lung injury model

All animal experiments were performed in accordance with institutional guidelines and approved by the relevant Animal Care and Use Committee. Acute lung injury was induced in mice by intratracheal instillation of lipopolysaccharide (LPS, 2.5 mg/kg body weight). 8-week-old mice (purchased from GemPharmatech, Nanjing, China) were anesthetized prior to the procedure, and LPS was administered in a defined volume of sterile phosphate-buffered saline (PBS). Mice were anesthetized before intratracheal instillation, and an equal volume of LPS solution was administered to each animal.

Monocyte-derived macrophages were generated *in vitro* and sorted into CD63^+^ and CD63^-^ populations by FACS-sorting. Sorted cells were washed and resuspended in sterile PBS. A defined number of macrophages (1x10^6^ cells per mouse) were delivered into recipient mice via intratracheal instillation at 24 hours after LPS challenge. Mice were monitored and sacrificed at 72 hours after cell transfer. Lung tissues were harvested for histological analysis, gene expression assessment, and flow cytometry-based immune cell profiling.

### Quantitative real-time PCR

Total RNA was isolated from *in vitro*-cultured macrophages and lung tissue-derived cells using TRIzol reagent (Invitrogen) according to the manufacturer’s instructions. Complementary DNA (cDNA) was synthesized using the TRUE script RT Master Mix (Aidlab, Cat#PC58). Quantitative real-time PCR (qRT-PCR) was performed using 2xSYBRGreenqPCRMix (Aidlab, Cat#PC3301). Gene expression levels were normalized to GAPDH or β-ACTINE. Gene-specific primer sequences are listed in **Table 1**.

**Table 1:** List of primer sequence, related to materials and methods.

Name	Forward (5’ to 3’)	Reverse (5’ to 3’)
*β-ACTIN*	CACCATTGGCAATGAGCGGTTC	AGGTCTTTGCGGATGTCCACGT
*GAPDH*	GTCTCCTCTGACTTCAACAGCG	ACCACCCTGTTGCTGTAGCCAA
*ELAVL1*	TGTTCTCTCGGTTTGGGCGGAT	TCTTCTGCCTCCGACCGTTTGT
*TNF*	CTCTTCTGCCTGCTGCACTTTG	ATGGGCTACAGGCTTGTCACTC
*IL1B*	CCACAGACCTTCCAGGAGAATG	GTGCAGTTCAGTGATCGTACAGG
*CXCL8*	GAGAGTGATTGAGAGTGGACCAC	CACAACCCTCTGCACCCAGTTT
*PFKL*	AAGAAGTAGGCTGGCACGACGT	GCGGATGTTCTCCACAATGGAC
*PGK1*	CCGCTTTCATGTGGAGGAAGAAG	CTCTGTGAGCAGTGCCAAAAGC
*SLC2A1*	TTGCAGGCTTCTCCAACTGGAC	CAGAACCAGGAGCACAGTGAAG
*LDHA*	GGATCTCCAACATGGCAGCCTT	AGACGGCTTTCTCCCTCTTGCT
*HIF1A*	TATGAGCCAGAAGAACTTTTAGGC	CACCTCTTTTGGCAAGCATCCTG
*CD206*	AGCCAACACCAGCTCCTCAAGA	CAAAACGCTCGCGCATTGTCCA
*CD163*	CCAGAAGGAACTTGTAGCCACAG	CAGGCACCAAGCGTTTTGAGCT
*RHOA*	TCTGTCCCAACGTGCCCATCAT	CTGCCTTCTTCAGGTTTCACCG
*CD63*	CAACCACACTGCTTCGATCCTG	GACTCGGTTCTTCGACATGGAAG
*ISG15*	CTCTGAGCATCCTGGTGAGGAA	AAGGTCAGCCAGAACAGGTCGT
*MX1*	GGCTGTTTACCAGACTCCGACA	CACAAAGCCTGGCAGCTCTCTA
*IFIT1*	GCCTTGCTGAAGTGTGGAGGAA	ATCCAGGCGATAGGCAGAGATC
*OAS1*	AGGAAAGGTGCTTCCGAGGTAG	GGACTGAGGAAGACAACCAGGT
*Il1b*	TGGACCTTCCAGGATGAGGACA	GTTCATCTCGGAGCCTGTAGTG
*Tnf*	GGTGCCTATGTCTCAGCCTCTT	GCCATAGAACTGATGAGAGGGAG
*Ccl2*	GCTACAAGAGGATCACCAGCAG	GTCTGGACCCATTCCTTCTTGG
*Il6*	TACCACTTCACAAGTCGGAGGC	CTGCAAGTGCATCATCGTTGTTC

### Hematoxylin and eosin staining

Hematoxylin and eosin (HE) staining was performed on mouse lung tissues. Briefly, lung tissues were fixed in 4% paraformaldehyde (PFA) for 4 h, followed by dehydration through a graded ethanol series (75%, 95%, and 100%). Samples were then embedded in paraffin and sectioned at 6 μm thickness. Paraffin sections were stained with hematoxylin and eosin (Beyotime Biotech, Cat#C0105M) according to standard protocols. Images were acquired using a Leica Aperio VERSA 8 whole-slide scanner, and histological analysis was performed using ImageScope (Leica).

### Cell sorting and flow cytometry analysis

For macrophage sorting, bone marrow-derived macrophages (BMDMs) generated from *in vitro* differentiation were stained with antibodies against macrophage surface markers and CD63. CD63^hi^ and CD63^lo^ macrophage populations were isolated by FACS-sorting under sterile conditions for downstream experiments. Flow cytometry analysis of lung-infiltrating neutrophils was performed on single-cell suspensions prepared from mouse lungs. Cells were resuspended in FACS buffer (PBS containing 1% BSA) and blocked with anti-mouse CD16/32 antibodies for 15 min prior to surface staining with antibodies against neutrophil markers. Cells were acquired on a FACSCelesta flow cytometer (BD Biosciences), and data were analyzed using FlowJo software. All antibodies used for flow cytometry and cell sorting are listed in **Table 2**.

**Table 2:** List of antibody, related to materials and methods.

Reagent or resource	Source	Identifier
Anti-mouse CD11b-Percp, (Clone M1/70)	Biolegend	Cat#101230;RRID: AB_2129374
Anti-mouse Ly6G-APC-cy7, (Clone 1A8)	Biolegend	Cat#127624;RRID: AB_10640819
Anti-mouse CD63-PE, (Clone NVG-2)	Biolegend	Cat#143903;RRID: AB_11203532
Anti-mouseCD16/32antibody, (Clone93)	Biolegend	Cat#101302;RRID: AB_312801

### Quantification and statistical analysis

Statistical analyses were performed using GraphPad Prism (v8.3.0) (https://www.graphpad.com/) and R (v4.2.1). Comparisons between two groups were conducted using unpaired two-tailed Student’s t-test, while multiple-group comparisons were analyzed by one-way analysis of variance (ANOVA). The number of biological replicates and specific statistical tests applied are indicated in the corresponding figure legends. Data are presented as mean ± standard deviation (SD) unless otherwise specified. Statistical significance was defined as follows: n.s., not significant; *, *p* < 0.05; **, *p* < 0.01; ***, *p* < 0.001; ****, *p* < 0.0001.

## Results

### Single-cell analysis reveals myeloid cell expansion and accumulation of monocyte-derived macrophages in COVID-19 lungs

**Figure 1:**
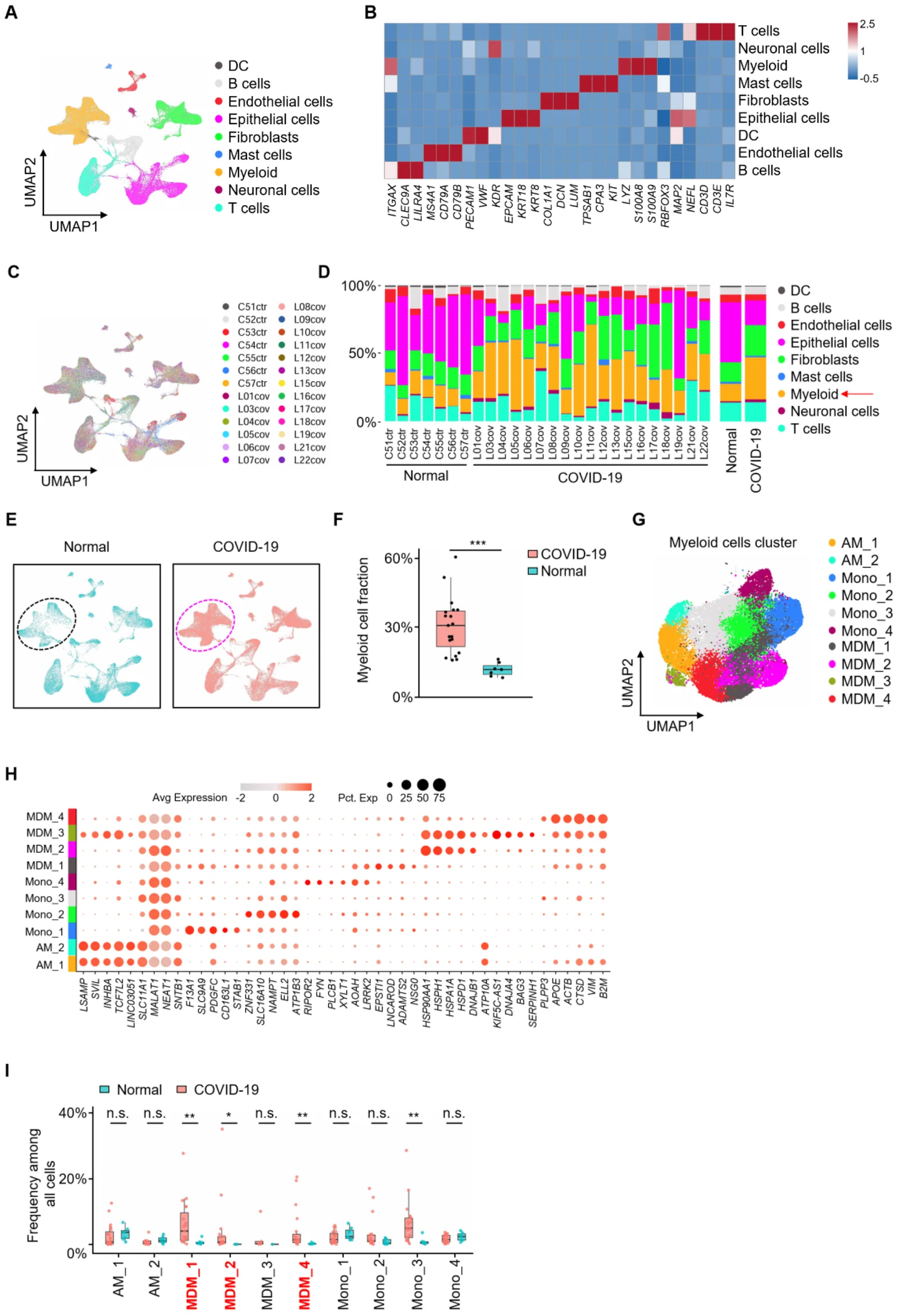
Single-cell characterization of lung myeloid cell heterogeneity in COVID-19. **(A)** UMAP visualization of major cell types in lung tissues from COVID-19 patients and control donors, based on Harmony-corrected PCA embeddings (see Materials and methods). **(B)** Heatmap showing the expression levels of canonical marker genes across the major cell types identified in **(A)**. **(C)** UMAP visualization of cellular distributions colored by individual patient origin, illustrating the contribution of each donor to the identified clusters and enabling assessment of inter-sample variability and batch integration. **(D)** Stacked bar plot showing the relative proportions of major cell types in lung tissues from COVID-19 patients and control donors. **(E)** UMAP visualization of major cell types in lung tissues from COVID-19 patients and control donors, with the myeloid compartment highlighted. **(F)** Proportion of myeloid cells among all profiled lung cells in COVID-19 and control samples (control donors, n = 7; COVID-19 patients, n = 19). **(G)** UMAP visualization of re-clustered myeloid populations, annotated as alveolar macrophages (AM_1-AM_2), monocytes (Mono_1-Mono_4), and monocyte-derived macrophages (MDM_1-MDM_4) for downstream analyses. **(H)** Bubble plot depicting the expression of representative marker genes across major myeloid cell subsets, illustrating their transcriptional identities. **(I)** Bar plot showing the proportional distribution of major myeloid cell types between COVID-19 patients and control donors, highlighting disease-associated shifts in myeloid composition. n.s., not significant; *p < 0.05; **p < 0.01; ***p < 0.001. [two-tailed Student’s t-test in **(F)** and one-way ANOVA analysis in **(I)**].

To determine whether COVID-19 infection reshapes the pulmonary immune landscape, we re-analyzed publicly available snRNA-seq datasets of lung tissues from COVID-19 patients and control individuals. After quality control filtering to remove low-quality nuclei, doublets, and cells with high mitochondrial gene content, the data were normalized and integrated into a unified analysis pipeline. In the integrated dataset, cells clustered according to intrinsic transcriptional identities rather than donor origin or disease status, indicating effective batch correction and reliable data integration (**Figure 1A**). Using canonical marker genes, we annotated major pulmonary cell populations, including epithelial cells, endothelial cells, fibroblasts, and multiple immune cell types such as T cells, B cells, dendritic cells, mast cells, and myeloid cells (**Figures 1A, B**). Each population displayed distinct and consistent marker expression patterns, supporting the biological interpretability of the atlas (**Figure 1B**). To further assess whether the integrated cellular landscape was influenced by donor-specific effects, we projected cells by individual patient identity onto the low-dimensional embedding. Cells derived from different donors were broadly intermingled rather than forming donor-specific clusters (**Figure 1C**), indicating that inter-individual variability did not dominate the clustering structure. This result further supports the effectiveness of batch correction and confirms that downstream analyses primarily capture biologically meaningful variation rather than technical or donor-driven biases.

We next examined whether COVID-19 infection alters the overall cellular composition of the lung. Comparative analysis revealed a pronounced remodeling of the cellular landscape, characterized by a marked expansion of myeloid cells accompanied by a relative depletion of structural cell populations, including epithelial cells (**Figure 1D**). This imbalance indicates extensive immune infiltration and disruption of tissue homeostasis during infection. Consistently, UMAP visualization demonstrated a clear enrichment of myeloid populations in COVID-19 lungs (**Figure 1E**), which was further confirmed by quantitative compositional analysis across samples (**Figure 1F**). Collectively, these results reveal that COVID-19 drives a global reorganization of the lung ecosystem, shifting it from a structurally balanced state toward an immune-dominant landscape, with myeloid cells representing a prominently expanded compartment.

Given the prominent expansion of myeloid cells in COVID-19 lungs, we next investigated whether this compartment harbors disease-associated cellular states. High-resolution re-clustering of myeloid cells from the integrated dataset revealed multiple transcriptionally distinct subsets (**Figure 1G**). Based on canonical marker genes and lineage-associated signatures, these subsets were classified into three major populations: alveolar macrophages (AM), monocytes (Mono), and monocyte-derived macrophages (MDM) (**Figures 1G, H**). Notably, the MDM compartment exhibited substantial transcriptional heterogeneity, suggesting the presence of functionally specialized subpopulations. To further define disease-associated subsets, we compared the relative abundance of each myeloid population between healthy controls and COVID-19 samples. This analysis identified several populations-predominantly within the MDM compartment-that were markedly enriched in COVID-19 samples (**Figure 1I**), highlighting a selective expansion of specific monocyte-derived macrophage states during infection. Together, these results indicate that monocyte-derived macrophages are markedly expanded in COVID-19 lungs and constitute a major contributor to the increased myeloid cell abundance.

### Identification of a CD63^hi^ inflammatory macrophage state within the monocyte-derived macrophage compartment

**Figure 2:**
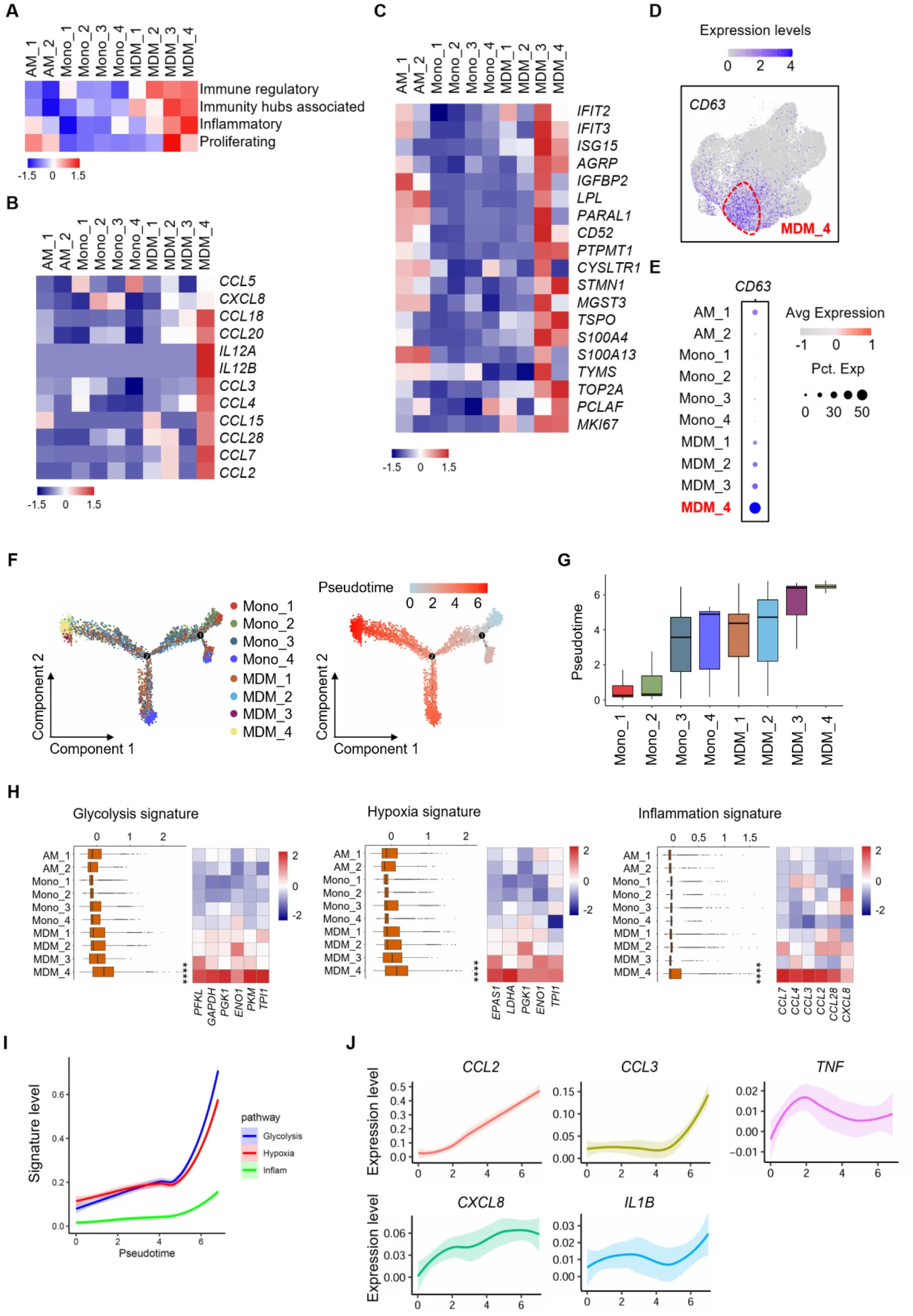
The transcriptional features and differentiation trajectory of myeloid cells. **(A)** Heatmap showing the expression of genes associated with immune regulatory, inflammatory, proliferative programs, and immunity hub-associated signatures across distinct myeloid cell populations. **(B)** Heatmap depicting the expression of inflammatory-related genes across different myeloid cell subsets. **(C)** Heatmap illustrating the expression of immunity hub-associated and proliferative signature genes across diverse myeloid cell populations. **(D)** UMAP visualization of *CD63* expression across myeloid cell populations, highlighting its enrichment within specific macrophage subsets (MDM_4). **(E)** Bubble plot showing *CD63* expression levels across myeloid cell populations, summarizing both expression magnitude and proportion of expressing cells. **(F)** Monocle2 pseudotime trajectory analysis depicting the differentiation trajectory from monocytes (Mono) to monocyte-derived macrophages (MDMs), with inferred pseudotime progression. **(G)** Boxplot illustrating the distribution of pseudotime values across monocyte and MDM populations, indicating progressive differentiation states. **(H)** Boxplot and heatmap showing enrichment scores of glycolysis, hypoxia, and inflammatory signatures across different myeloid cell populations. **(I)** Line plot depicting dynamic changes in glycolysis, hypoxia, and inflammatory gene signature scores along pseudotime progression. **(J)** Line plot showing the temporal expression dynamics of pro-inflammatory genes along the inferred pseudotime trajectory. ****p < 0.0001 [One-way ANOVA analysis in **(H)**].

Given the marked expansion of monocyte-derived macrophages (MDMs) in COVID-19 lungs, we next sought to further resolve their functional heterogeneity. To this end, we re-analyzed the transcriptional states of MDMs using a functional classification framework. MDMs were stratified into four major states based on previously reported marker gene signatures defining distinct macrophage functional programs, including immune regulatory macrophages (*CD163*, *CD206*, *SPP1*, *RHOA*, *HLA-A*, *AZIN1*, *TUBA1A*), immunity hub-associated macrophages (*IFIT2*, *IFIT3*, *ISG15*, *AGRP*, *IGFBP2*, *LPL*, *PARAL1*), inflammatory cytokine-enriched macrophages (*FCN1*, *MAFF*, *KLF4*, *NFE2L2*, *BACH1*), and proliferating macrophages (*TYMS*, *STMN1*, *TOP2A*, *PCLAF*, *MKI67*). (**Figure 2A**). Hierarchical clustering showed that MDMs did not segregate into discrete clusters but instead formed a continuous transcriptional spectrum, suggesting progressive functional adaptation during infection (**Figure 2A**). Notably, MDM_1 corresponded to immunity hub-associated macrophages, whereas MDM_2 aligned with immune regulatory macrophages. MDM_3 exhibited a hybrid phenotype with both immunity hub-associated and proliferative features, while MDM_4 was predominantly defined by inflammatory cytokine-enriched signatures (**Figure 2A**). Consistent with these assignments, MDM_4 showed a canonical inflammatory profile characterized by high expression of chemokines and inflammatory mediators, including *CCL2*, *CCL3*, *CCL4*, and *CCL18* (**Figure 2B**), whereas MDM_3 was enriched for interferon-stimulated and cell cycle-related gene programs (**Figure 2C**).

Notably, the inflammatory cytokine-enriched MDM_4 population is of particular interest in COVID-19 due to its strong pro-inflammatory transcriptional program. However, surface markers defining inflammatory macrophage states remain incompletely characterized. To address this, we systematically examined candidate marker expression across MDM compartments. Importantly, *CD63* was selectively and highly enriched in the MDM_4 compartment, identifying CD63^hi^ macrophages as corresponding to the inflammatory cytokine-enriched state (**Figures 2D, E**). Consistently, CD63^hi^ macrophages were significantly increased in COVID-19 lungs compared with healthy controls (**Figure 1I**), indicating a disease-associated amplification of this inflammatory macrophage program.

To further characterize state transitions, pseudotime trajectory analysis of monocytes and MDM subpopulations revealed a continuous progression from immune regulatory and immunity hub-associated states (MDM_1-MDM_3) toward inflammatory cytokine-enriched macrophage states (CD63^hi^-MDM_4) (**Figures 2F, G**). This transition was accompanied by progressive activation of inflammatory signaling pathways, along with coordinated metabolic and microenvironmental adaptations, including enhanced glycolytic activity and hypoxia-associated transcriptional responses (**Figures 2H, I**), collectively delineating a coordinated inflammatory and metabolic reprogramming of macrophage states during infection. We next examined the dynamic expression patterns of canonical inflammatory mediators along pseudotime. Notably, key pro-inflammatory cytokines and chemokines, including *CCL2*, *CCL3*, *CXCL8*, *IL1B*, and *TNF*, exhibited a progressive increase in expression as cells transitioned toward the CD63^hi^ macrophage state (**Figure 2J**). Collectively, these findings identify CD63^hi^ macrophages as a recurrent inflammatory state within the monocyte-derived macrophage compartment across COVID-19 lung tissues. This CD63^hi^ population corresponds to the MDM_4 inflammatory program and represents a terminally activated macrophage state characterized by sustained inflammatory signaling, together with enhanced glycolytic activity and hypoxia-associated transcriptional responses, suggesting that the surface protein CD63 can be used to characterize this inflammatory macrophage state.

### CD63^hi^ macrophages represent a lung-enriched inflammatory state across independent COVID-19 datasets

**Figure 3:**
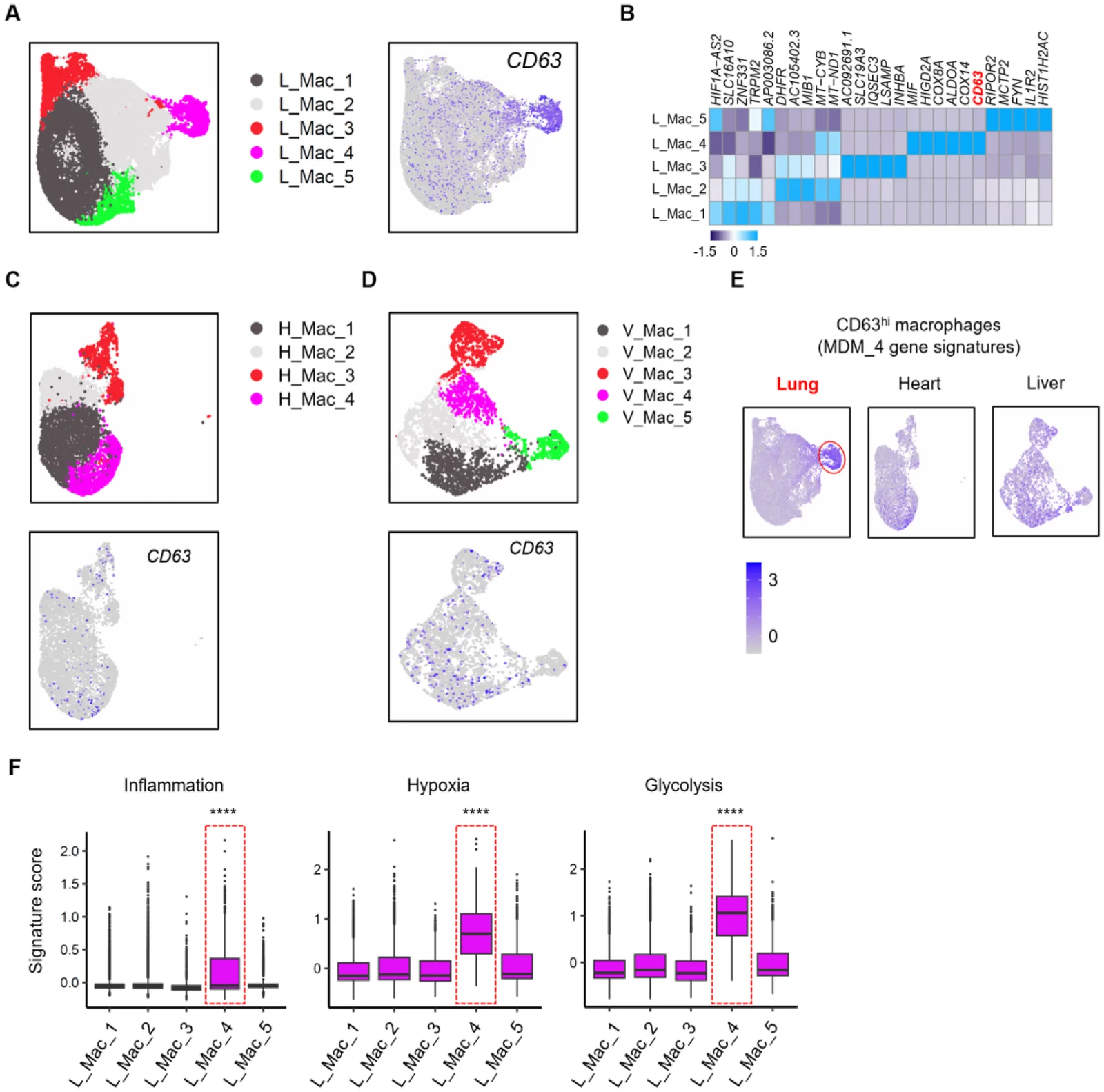
Tissue distribution and transcriptional features of CD63^hi^ macrophages across multiple organs in COVID-19. **(A)** Left: UMAP visualization of myeloid-derived macrophages from lung tissues of COVID-19 patients, identifying five subclusters (L_Mac_1-L_Mac_5). Right: Feature plot showing *CD63* expression across lung macrophage subsets. **(B)** Heatmap depicting the expression of representative marker genes defining each lung macrophage subpopulation identified in **(A)**. **(C)** Top: UMAP visualization of cardiac-resident macrophages from COVID-19 patients, identifying four subclusters (H_Mac_1-H_Mac_4). Bottom: Feature plot showing *CD63* expression across cardiac macrophage subsets. **(D)** Top: UMAP visualization of hepatic macrophages from COVID-19 patients, identifying five subclusters (V_Mac_1-V_Mac_5). Bottom: Feature plot showing *CD63* expression across hepatic macrophage subsets. **(E)** UMAP visualization of CD63^hi^ macrophage signature gene expression across lung-, heart-, and liver-derived macrophage populations in COVID-19 patients, with CD63^hi^ macrophages specifically enriched in lung tissues highlighted. **(F)** Boxplot showing the expression levels of inflammatory, hypoxia-related, and glycolytic gene signatures across distinct lung macrophage subpopulations in COVID-19 patients. ****p < 0.0001 [One-way ANOVA analysis in **(F)**].

To validate the robustness of CD63^hi^ macrophages across independent datasets, we re-analyzed an independent single-cell RNA-seq dataset of COVID-19 lung tissues (Delorey et al., 2021). Unsupervised clustering of macrophages revealed a distinct CD63^hi^ subpopulation, with UMAP visualization demonstrating selective and high CD63 expression within this cluster (**Figures 3A, B**). To further assess the tissue specificity of this CD63^hi^ macrophage state, we next examined infiltrating macrophages from heart and liver within the same dataset. In contrast to lung tissues, macrophages from both heart and liver exhibited low CD63 expression, and no comparable CD63^hi^ subpopulation was observed in these organs (**Figures 3C, D**), suggesting that this macrophage state is selectively enriched in the lung microenvironment during infection. We then performed module scoring analysis using the CD63^hi^ macrophage signature genes across macrophages from lung, heart, and liver tissues. The CD63^hi^ signature was specifically and strongly enriched in lung macrophages, whereas no significant enrichment was detected in cardiac or hepatic macrophages (**Figure 3E**), further supporting the lung-restricted nature of this inflammatory macrophage state.

Functional characterization of macrophage subclusters within this independent lung dataset revealed that the L_Mac_4 population, corresponding to CD63^hi^ macrophages, consistently exhibited elevated expression of inflammatory genes, as well as hypoxia- and glycolysis-associated transcriptional programs (**Figure 3F**). These results were highly consistent with our previous findings, indicating that *CD63* marks a reproducible inflammatory macrophage state characterized by coordinated inflammatory and metabolic reprogramming, which is selectively enriched in COVID-19 lung tissues across independent datasets.

### Dynamic activation of JUND is associated with the transition toward the CD63^hi^ inflammatory macrophage state

**Figure 4:**
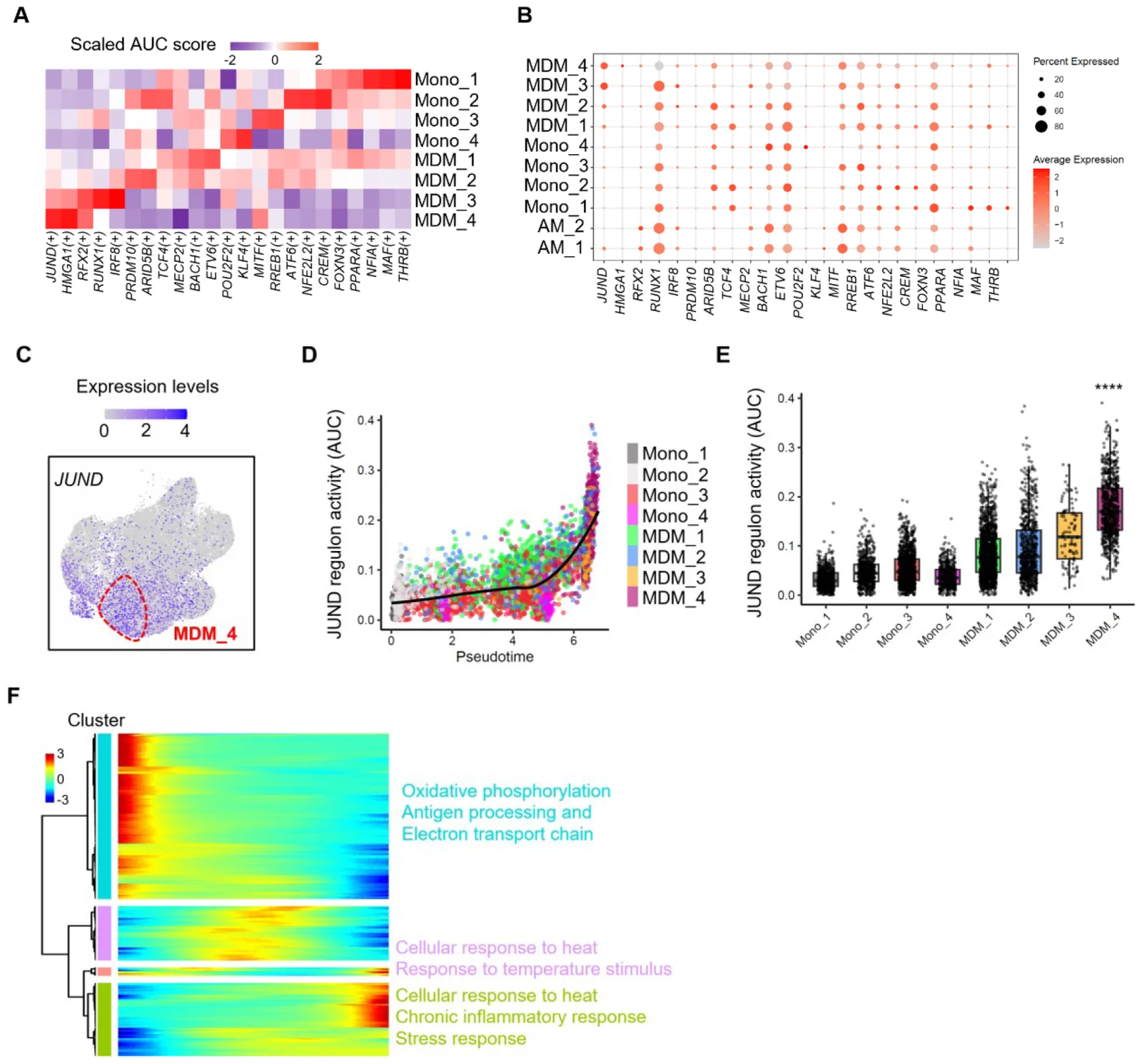
Transcriptional regulatory dynamics underlying CD63^hi^ macrophage differentiation along the monocyte-to-MDM trajectory. **(A)** Transcription factor activity profiling across AMs, monocyte and monocyte-derived macrophage (MDM) subsets. **(B)** Bubble plot showing the expression levels of the transcription factors identified in **(A)** across major myeloid cell populations. **(C)** UMAP visualization of *JUND* expression across different myeloid cell subsets. **(D)** Distribution of *JUND* regulon activity along pseudotime across the monocyte-to-MDM differentiation trajectory. **(E)** Boxplot showing *JUND* regulon activity across distinct myeloid cell populations. **(F)** Heatmap showing the dynamic changes of gene expression along pseudotime. The differentially expressed genes were clustered hierarchically into three groups, and the representative enriched pathways of each group were shown. ****p < 0.0001 [One-way ANOVA analysis in **(E)**].

To identify regulators associated with macrophage state transitions toward the CD63^hi^ inflammatory phenotype, we investigated transcriptional regulatory dynamics along the monocyte-to-MDM continuum. Systematic screening of transcriptional regulators based on activity patterns across monocyte and MDM subsets identified a subset of candidates, including *JUND* and *HMGA1*, that exhibited preferential activity in CD63^hi^ macrophages (MDM_4), as reflected by elevated AUC scores in this population relative to other subsets (**Figure 4A**). Consistently, expression analysis revealed a progressive increase of *JUND* along the monocyte-to-MDM trajectory, with peak expression observed in the inflammatory MDM_4 state (**Figures 4B, C**). Projection of regulator activity onto the pseudotime trajectory further showed that *JUND* activity gradually increased and reached maximal levels at the terminal CD63^hi^ state (**Figures 4D, E**), suggesting that increased *JUND* activity is associated with the acquisition of the CD63^hi^ inflammatory macrophage state.

To further characterize transcriptional programs underlying this trajectory, we performed GO enrichment analysis across differentiation states, which revealed distinct functional modules associated with early, intermediate, and late stages. Early-stage modules were enriched in oxidative phosphorylation, electron transport chain activity, and antigen processing pathways, indicative of a metabolically active and immune surveillance-oriented state (**Figure 4F**). Intermediate-stage programs were characterized by stress-responsive pathways, including cellular responses to heat and temperature stimuli, consistent with a transitional stress-adaptive state (**Figure 4F**). In contrast, late-stage branches corresponding to CD63^hi^ macrophages were strongly enriched in cellular stress responses and chronic inflammatory signaling pathways, reflecting a terminal inflammatory state marked by sustained stress signaling and inflammatory reprogramming (**Figure 4F**).

Taken together, these results delineate a progressive transcriptional reprogramming of monocyte-derived macrophages along the differentiation trajectory, culminating in a CD63^hi^ inflammatory state. Within this continuum, elevated JUND activity characterizes the late-stage CD63^hi^ macrophage state, suggesting its potential association with inflammatory macrophage remodeling.

### *ELAVL1* regulates inflammatory and metabolic reprogramming of CD63^hi^ macrophages during macrophage state transitions

**Figure 5:**
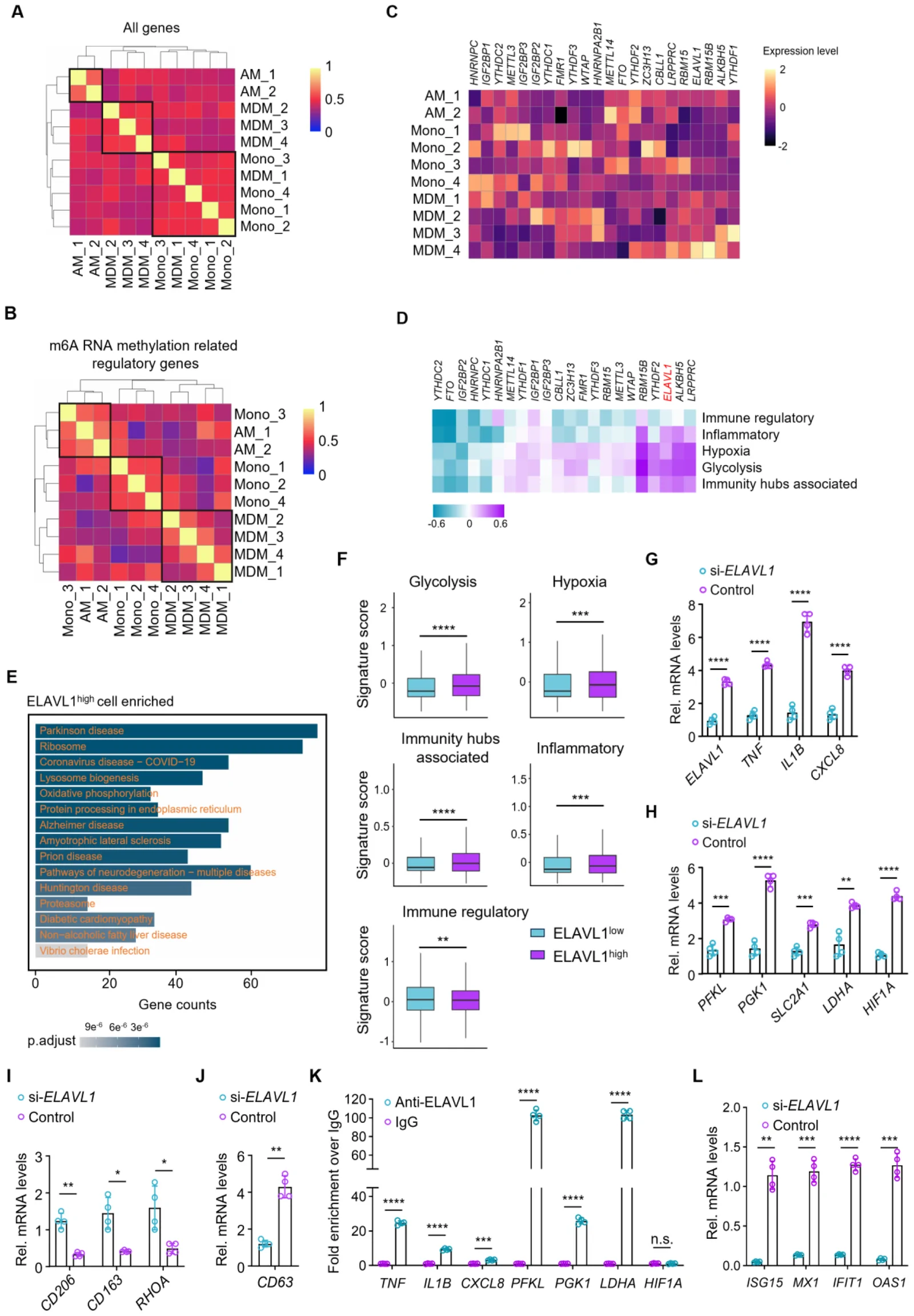
*ELAVL1* regulates inflammatory and metabolic reprogramming during macrophage state transitions. **(A)** Comparison of Unsupervised clustering results of alveolar macrophages (AM_1-AM_2), monocytes (Mono_1-Mono_4), and monocyte-derived macrophages (MDM_1-MDM_4) based on all genes. **(B)** Comparison of Unsupervised clustering results of alveolar macrophages (AM_1-AM_2), monocytes (Mono_1-Mono_4), and monocyte-derived macrophages (MDM_1-MDM_4) based on m^6^A RNA methylation related regulatory genes. **(C)** Heatmap showing the expression of m^6^A-associated regulatory genes between alveolar macrophages, monocytes, and monocyte-derived macrophages. **(D)** Correlation analysis between *ELAVL1* expression and macrophage functional signatures, including immune regulatory, inflammatory, hypoxia-, glycolysis-, and immunity hub-associated programs. **(E)** GO gene set enrichment analysis of biological processes enriched in *ELAVL1*^high^ cells. **(F)** Comparison of functional signature scores between *ELAVL1*^high^ and *ELAVL1*^low^ monocyte-derived macrophages, including inflammatory, glycolysis-, hypoxia-, immunity hub-associated, and immune regulatory programs. **(G)** qPCR analysis of *ELAVL1* knockdown efficiency and expression levels of representative pro-inflammatory genes (*TNF*, *IL1B*, *CXCL8*) in monocyte-derived macrophages following inflammatory stimulation in si-ELAVL1 and control groups. **(H)** qPCR analysis of glycolysis- and hypoxia-related genes (*PFKL*, *PGK1*, *SLC2A1*, *LDHA*, *HIF1A*) in monocyte-derived macrophages following *ELAVL1* knockdown (si-*ELAVL1*) compared with control. **(I)** qPCR analysis of immune regulatory markers (*CD206*, *CD163*, *RHOA*) in monocyte-derived macrophages following *ELAVL1* knockdown (si-*ELAVL1*) compared with control. **(J)** qPCR analysis of *CD63* expression in monocyte-derived macrophages following *ELAVL1* knockdown (si-*ELAVL1*) compared with control. **(K)** Binding of ELAVL1 to target genes in monocyte-derived macrophages, analyzed by RNA immunoprecipitation-qPCR. **(L)** qPCR analysis of interferon-associated gene expression in monocyte-derived macrophages. n.s., not significant; *p < 0.05; **p < 0.01; ***p < 0.001; ****p < 0.0001 [two-tailed Students’ t test in **(G–L)**].

Together with the transcriptional regulator *JUND* identified above, these findings suggest that macrophage inflammatory state transitions may be coordinately shaped by both transcriptional and epitranscriptomic regulatory layers. To explore whether epitranscriptomic regulation contributes to macrophage state transitions from immune regulatory to terminal inflammatory programs, we focused on N6-methyladenosine (m^6^A), the most abundant internal mRNA modification. m^6^A-associated regulators are known to modulate RNA stability and translation efficiency, thereby influencing immune activation and cellular state transitions (Fang et al., 2025; Feng et al., 2026; Sun et al., 2025). However, whether such regulatory programs reflect macrophage lineage identity or functional states in COVID-19 remains unclear. To address this, we first performed unsupervised clustering based on the global transcriptome, which robustly separated monocyte-derived macrophage subsets and alveolar macrophages (AM), reflecting clear lineage organization within the macrophage compartment (**Figure 5A**). We then examined macrophage organization based solely on m^6^A regulator expression. Notably, m^6^A-based clustering also preserved distinct separation between AM and MDM populations (**Figure 5B**), indicating that m^6^A-associated regulatory signatures retain lineage-related information in addition to potential state-dependent variation.

At the expression level, individual m^6^A-associated regulators exhibited heterogeneous distribution patterns across macrophage subsets (**Figure 5C**), suggesting partial functional specialization within distinct macrophage states. To identify candidate regulators linked to macrophage functional remodeling, we performed correlation analysis and identified *ELAVL1* as a prominent candidate. *ELAVL1* showed a negative association with immune regulatory macrophage programs, while displaying positive correlations with inflammatory, hypoxia-, glycolysis-, and immunity hub-associated signatures (**Figure 5D**), suggesting its potential involvement in macrophage polarization toward inflammatory states. Consistently, stratification of MDMs based on *ELAVL1* expression revealed that *ELAVL1*^high^ cells were enriched for pathways related to protein synthesis and cellular stress responses, including ribosome function, lysosome biogenesis, and protein processing in the endoplasmic reticulum, as well as metabolic pathways such as oxidative phosphorylation (**Figure 5E**), indicative of a stress-adapted and metabolically active state. In parallel, *ELAVL1*^high^ macrophages exhibited elevated inflammatory, glycolysis-, hypoxia-, and immunity hub-associated programs, whereas immune regulatory signatures were reduced (**Figure 5F**), supporting a shift toward an inflammatory phenotype. Although enrichment of neurodegeneration-associated pathways was also observed, these likely reflect shared stress and proteostasis-related processes rather than tissue-specific neuronal functions.

To functionally assess the role of ELAVL1 in macrophage state regulation, we established an *in vitro* macrophage polarization system and performed *ELAVL1* knockdown in monocyte-derived macrophages. *ELAVL1* silencing was confirmed at the mRNA level (**Figure 5G**). Upon LPS and IFN-γ stimulation, *ELAVL1*-depleted macrophages exhibited a marked reduction in pro-inflammatory gene expression, including *TNF*, *IL1B* and *CXCL8* (**Figure 5G**). Concurrently, key glycolysis- and hypoxia-associated genes, such as *PFKL*, *PGK1*, *SLC2A1*, *LDHA*, and *HIF1A*, were also significantly downregulated, indicating impaired acquisition of a metabolically reprogrammed inflammatory state (**Figure 5H**). In contrast, expression of immune regulatory markers, including *CD206, CD163*, and *RHOA*, was relatively increased in *ELAVL1*-deficient macrophages (**Figure 5I**), suggesting a partial shift toward an immune regulatory phenotype. Consistently, *CD63* expression was reduced following *ELAVL1* knockdown (**Figure 5J**), further supporting its association with the inflammatory macrophage state.

We next performed RNA immunoprecipitation followed by quantitative PCR (RIP-qPCR) to examine the association of ELAVL1 with transcripts involved in CD63^hi^ macrophage-associated functional programs. ELAVL1 immunoprecipitation demonstrated significant enrichment of multiple inflammatory and glycolytic transcripts, including *TNF*, *IL1B*, *CXCL8*, *PFKL*, *PGK1*, and *LDHA*, compared with IgG controls, indicating that ELAVL1 physically associates with mRNAs involved in inflammatory activation and metabolic reprogramming (**Figure 5K**). However, *HIF1A* showed no significant enrichment in ELAVL1 immunoprecipitates, suggesting that the regulation of HIF1A-associated hypoxia responses may occur indirectly through ELAVL1-associated regulatory networks rather than direct RNA binding (**Figure 5K**). These findings provide additional molecular evidence supporting the contribution of ELAVL1 to inflammatory and metabolic remodeling in CD63^hi^ macrophage-associated states. Because CD63^hi^ macrophages also displayed enrichment of interferon-associated antiviral signatures, we further examined whether ELAVL1 contributes to regulation of this transcriptional program. Following LPS stimulation, *ELAVL1*-deficient macrophages exhibited reduced expression of interferon-stimulated genes, including *ISG15*, *MX1*, *OAS1*, and *IFIT1* (**Figure 5L**).

Collectively, these findings indicate that *ELAVL1* contributes to the maintenance of inflammatory, interferon-responsive, and metabolically reprogrammed macrophage programs, and that *ELAVL1* depletion partially attenuates CD63^hi^ macrophage-associated inflammatory features while promoting a more immune-regulatory phenotype.

### CD63^hi^ macrophages define a conserved inflammatory program associated with disease severity and drive lung inflammation *in vivo*

**Figure 6:**
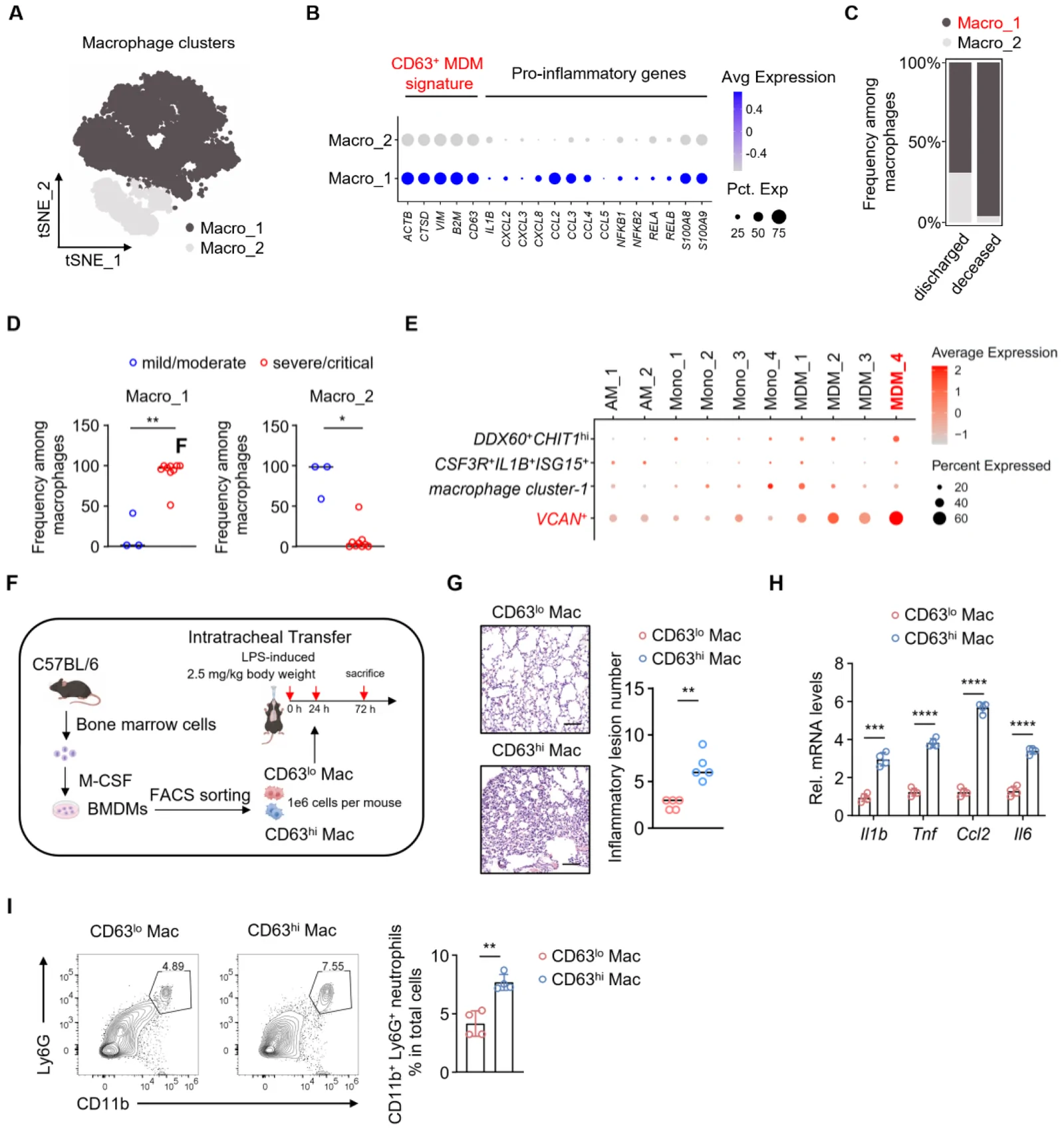
CD63^hi^ macrophages are associated with COVID-19 severity and promote lung inflammation and injury. **(A)** Re-clustering of macrophage populations from an independent single-cell RNA-seq dataset derived from bronchoalveolar lavage fluid (BALF) of COVID-19 patients. **(B)** Feature plots showing the expression of CD63^hi^ macrophage signature genes and representative inflammatory genes across BALF-derived macrophage subsets (Macro_1 and Macro_2). **(C)** Comparison of Macro_1 and Macro_2 proportions between deceased and discharged COVID-19 patients. **(D)** Proportion of Macro_1 and Macro_2 across different disease severity groups (mild/moderate, n = 3; severe/critical, n = 9). **(E)** Comparative analysis of macrophage subsets identified in this study with previously reported inflammatory macrophage populations from independent COVID-19 single-cell transcriptomic studies. **(F)** Schematic illustration of the experimental design for adoptive transfer of *in vitro*-differentiated CD63^hi^ and CD63^lo^ monocyte-derived macrophages into an LPS-induced acute lung injury mouse model. **(G)** Histological analysis and quantitative assessment of lung injury following transfer of CD63^hi^ versus CD63^lo^ macrophages; scale bars, 100 um. **(H)** qPCR analysis of pro-inflammatory cytokine expression (*Il1b*, *Tnf*, *Ccl2*, *Il6*) in lung tissues following macrophage transfer (n = 4 per group). **(I)** Flow cytometry analysis of CD11b^+^Ly6G^+^ neutrophil proportions in lung tissues following macrophage transfer (n = 4 per group). *p < 0.05; **p < 0.01; ***p < 0.001; ****p < 0.0001; [two-tailed Students’ t test in **(D, G–I)**].

To assess the reproducibility of the CD63^hi^ inflammatory macrophage state across anatomical compartments, we analyzed an independent single-cell RNA-seq dataset derived from bronchoalveolar lavage fluid (BALF) of COVID-19 patients (Ren et al., 2021). Re-clustering of macrophage populations identified two major subsets with stable transcriptional profiles (**Figures 6A, B**). Among these, one subset (Macro_1) closely recapitulated the CD63^hi^ macrophage population identified in lung tissue. At the transcriptional level, Macro_1 exhibited strong expression of the CD63^hi^ macrophage signature along with a broad panel of pro-inflammatory genes, including *CCL2*, *CCL3*, and *CXCL8* (**Figure 6B**), indicating that CD63^hi^ macrophages are reproducibly detected across lung parenchyma and airway compartments.

We next investigated the clinical relevance of this macrophage state. CD63^hi^ macrophages (Macro_1) were markedly enriched in deceased patients compared with discharged individuals (**Figure 6C**). In addition, stratification by disease severity revealed a progressive increase in the proportion of CD63^hi^ macrophages (Macro_1) in severe and critical cases relative to mild or moderate disease (**Figure 6D**), demonstrating a strong association between this macrophage state and adverse clinical outcomes. These results indicate that CD63^hi^ macrophages represent a clinically relevant inflammatory activation state closely linked to COVID-19 severity.

To further define the relationship between CD63^hi^ macrophages and previously reported inflammatory macrophage populations, we compared CD63^hi^ MDMs with published COVID-19-associated macrophage signatures, including *DDX60*^+^*CHIT1*^hi^, *CSF3R*^+^*IL1B*^+^*ISG15*^+^, macrophage cluster-1, and *VCAN*^+^ macrophage signatures (Lee et al., 2021; Shaath et al., 2020; Wang et al., 2024). CD63^hi^ MDMs showed the strongest enrichment for the *VCAN*^+^ macrophage signature, whereas relatively weaker similarity was observed with other reported inflammatory macrophage populations (**Figure 6E**). These results indicate that CD63^hi^ MDMs represent a monocyte-derived inflammatory macrophage state closely related to previously identified pathogenic macrophage populations in severe COVID-19, while retaining a distinct transcriptional identity.

To determine whether CD63^hi^ macrophages actively contribute to lung inflammation, we established a murine model of LPS-induced acute lung injury. Monocyte-derived macrophages were differentiated *in vitro* and stratified into CD63^hi^ and CD63^lo^ populations prior to intratracheal transfer into LPS-challenged mice (**Figure 6F**). Histological analysis revealed that mice receiving CD63^hi^ macrophages exhibited markedly exacerbated lung injury, including increased alveolar wall thickening and enhanced immune cell infiltration, compared with those receiving CD63^lo^ macrophages (**Figure 6G**).

Consistently, expression of key pro-inflammatory mediators, including *Il1b*, *Tnf*, *Ccl2*, and *Il6*, was significantly elevated in lungs of mice receiving CD63^hi^ macrophages (**Figure 6H**), indicating amplification of local inflammatory responses. In parallel, immune profiling demonstrated a pronounced increase in neutrophil (Ly6G^+^) accumulation following CD63^hi^ macrophage transfer (**Figure 6I**), reflecting a reinforced pro-inflammatory immune microenvironment.

**Figure 7:**
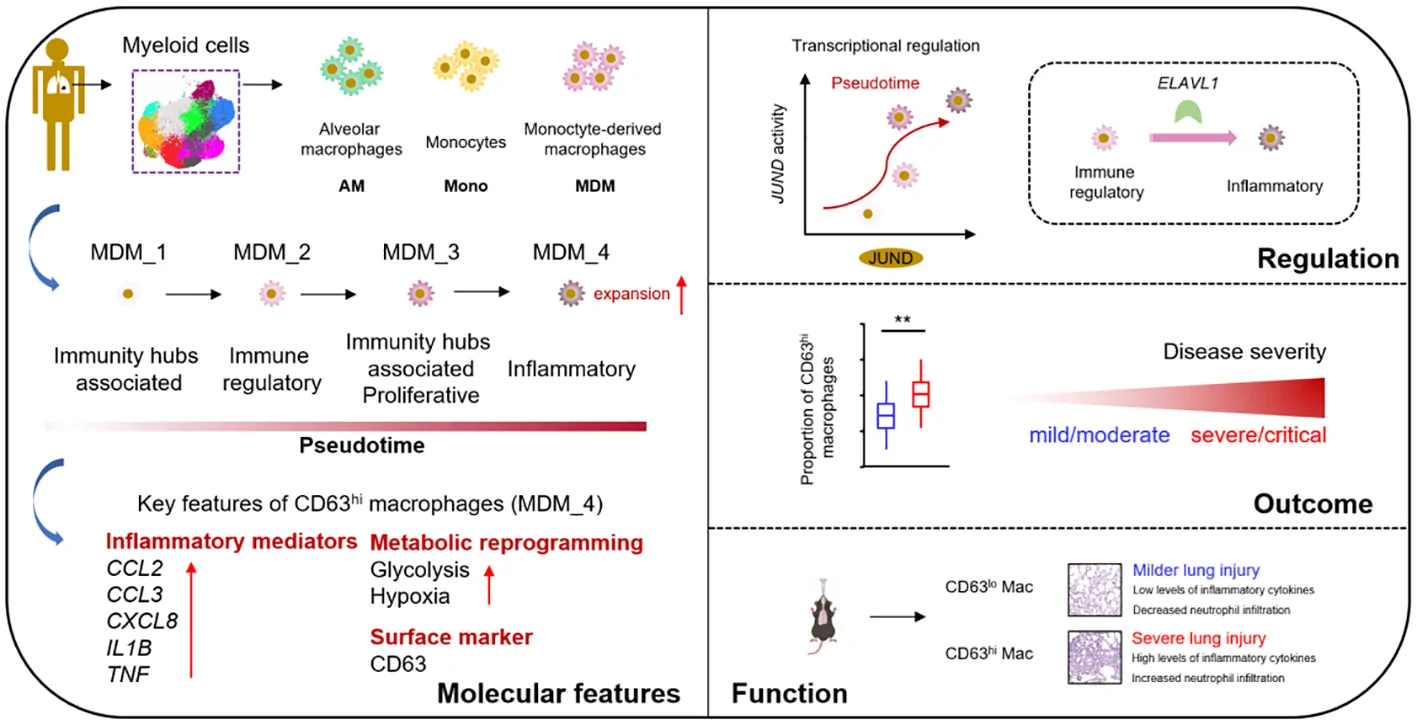
Schematic summary illustrating a lung-enriched CD63^hi^ terminal inflammatory macrophage state that amplifies lung inflammation and is associated with COVID-19 severity.

Collectively, these results demonstrate that CD63^hi^ monocyte-derived macrophages represent a conserved inflammatory activation state across datasets and compartments, are strongly associated with disease severity, and functionally exacerbate lung inflammation and injury *in vivo* (**Figure 7**).

## Discussion

Immune dysregulation in the lung is a hallmark of severe COVID-19, yet the cellular states and regulatory mechanisms driving pathogenic inflammation remain incompletely defined. Although macrophage inflammatory programs have been widely reported, their phenotypic definitions are often inconsistent across studies (Bailey et al., 2024; Merad and Martin, 2020; Yao et al., 2020; Zhang et al., 2021). In particular, while monocyte-derived macrophage (MDM) expansion is well documented, the identity of pathogenic macrophage states and their molecular regulators remains unresolved. Here, we identify a distinct CD63^hi^ macrophage population that is markedly expanded in COVID-19 lungs and represents a conserved terminal inflammatory fate within the monocyte-derived macrophage compartment. Rather than reflecting a transient activation state, CD63^hi^ macrophages exhibit coordinated activation of inflammatory transcriptional programs coupled with glycolytic and hypoxia-associated metabolic reprogramming, consistent with a stable inflammatory fate attractor within the macrophage differentiation landscape. Trajectory analysis further supports a progressive transition from immune-regulatory and intermediate macrophage states toward this CD63^hi^ endpoint, suggesting that severe inflammatory microenvironments actively reshape macrophage fate decisions toward a terminally committed inflammatory state.

Importantly, CD63^hi^ macrophages are reproducibly detected across independent single-cell datasets and anatomical compartments, including lung parenchyma and bronchoalveolar lavage fluid, indicating that they represent a conserved inflammatory state rather than a dataset-specific annotation. Despite variability in patient cohorts and sampling strategies, these cells consistently exhibit enrichment of cytokine production, stress response, and metabolic reprogramming pathways. This convergence supports the existence of a shared macrophage differentiation program underlying severe COVID-19, consistent with emerging models of context-dependent macrophage state plasticity in inflammatory disease.

Functionally, CD63^hi^ macrophages exhibit strong pathogenic capacity *in vivo*. Adoptive transfer into a murine model of acute lung inflammation exacerbates tissue injury, amplifies pro-inflammatory cytokine production, and promotes neutrophil infiltration, demonstrating that this population actively drives immunopathology rather than merely reflecting inflammatory burden. Although this inflammatory model does not fully recapitulate SARS-CoV-2 infection, it provides a functional framework to assess the tissue-damaging potential of macrophage states identified from human COVID-19 lungs. These findings place CD63^hi^ macrophages as a functionally dominant effector state contributing to tissue damage amplification in the inflamed lung microenvironment. Notably, although CD63^hi^ macrophages exhibit features characteristic of severe COVID-19-associated immune remodeling, including interferon-associated antiviral programs, several core features of this state, such as NF-κB activation, inflammatory cytokine production, and glycolytic reprogramming, are shared with other severe inflammatory lung conditions. Therefore, CD63^hi^ macrophages are best interpreted as a context-dependent terminal inflammatory macrophage state rather than a COVID-19-exclusive lineage. Mechanistically, our data reveal a hierarchical regulatory architecture integrating transcriptional and post-transcriptional programs that govern CD63^hi^ macrophage fate determination. At the transcriptional level, the AP-1 family transcription factor JUND shows progressive activation along the monocyte-to-MDM differentiation trajectory, reaching maximal activity in CD63^hi^ macrophages, suggesting a role in enforcing late-stage inflammatory differentiation. This aligns with previous reports implicating AP-1 signaling in macrophage inflammatory polarization and stress-adaptive transcriptional responses (Tseng et al., 2022; Wan et al., 2022; Zhao et al., 2024). Previous studies have shown that JUND regulates macrophage activation, oxidative stress responses, and IL-1β-associated inflammatory signaling (Behmoaras et al., 2008; Hull et al., 2013). Therefore, persistent inflammatory and stress-associated cues within the severe COVID-19 lung microenvironment may sustain JUND-associated transcriptional activity, thereby contributing to the maintenance of a maladaptive inflammatory macrophage state. Such stress-responsive transcriptional reinforcement may cooperate with NF-κB signaling and metabolic remodeling to amplify cytokine production and tissue injury, consistent with the hyperinflammatory macrophage activation observed in severe COVID-19 (Laurent et al., 2022; McGonagle et al., 2020).

In parallel, we identify *ELAVL1* as a central post-transcriptional regulatory hub coordinating inflammatory and metabolic reprogramming. *ELAVL1* expression positively correlates with inflammatory, glycolytic, and hypoxia-associated pathways, while inversely correlating with immune-regulatory signatures, suggesting its role in stabilizing pro-inflammatory gene expression programs. Functional perturbation experiments demonstrate that *ELAVL1* depletion suppresses inflammatory cytokine production, attenuates metabolic activation, and biases macrophages toward an immune-regulatory phenotype, accompanied by reduced *CD63* expression. However, our current data do not establish ELAVL1 as a direct determinant of the CD63^hi^ macrophage phenotype or demonstrate CD63 as a direct downstream target of ELAVL1. Instead, ELAVL1 likely participates in a broader RNA regulatory network that modulates inflammatory and metabolic programs associated with this macrophage state. Consistent with the reduction of glycolytic and hypoxia-associated programs after ELAVL1 depletion, *HIF1A* expression was decreased, suggesting that ELAVL1-associated RNA regulatory activity may contribute to metabolic adaptation in inflammatory macrophages. Although *HIF1A* was not identified as a direct ELAVL1-bound transcript in our RIP-qPCR analysis, previous studies have suggested that ELAVL1 may influence hypoxia responses through indirect post-transcriptional regulatory networks, including modulation of HIF1A-associated pathways (Xie et al., 2024). These findings support a model in which ELAVL1-associated post-transcriptional regulation contributes to the maintenance of inflammatory macrophage programs by coordinating RNA regulatory processes with metabolic adaptation. Together with the observed increase in *JUND* activity along the monocyte-to-MDM trajectory, these findings suggest a potential multilayer regulatory framework associated with the establishment of the CD63^hi^ inflammatory macrophage state.

Previous studies have highlighted the importance of macrophage heterogeneity and metabolic reprogramming in inflammatory diseases (Chavakis et al., 2023; Heieis et al., 2023; Houston, 2023). Our findings extend these observations by defining a surface marker-associated inflammatory fate state and linking it to an integrated transcriptional-epitranscriptomic regulatory axis. Notably, CD63, a member of the tetraspanin family, serves here as a surface marker defining a distinct inflammatory macrophage state rather than a demonstrated upstream driver of macrophage polarization. CD63 is primarily localized to late endosomal and lysosomal compartments and is highly enriched in extracellular vesicles, where it participates in endosomal trafficking, vesicle biogenesis, and intercellular communication (Federici et al., 2020; Levy et al., 1998; Pols and Klumperman, 2009). Therefore, elevated CD63 expression in CD63^hi^ macrophages may reflect enhanced endosomal-lysosomal activity and vesicle-associated immune regulation accompanying inflammatory activation. Recent studies have highlighted the involvement of extracellular vesicles in COVID-19-associated immune dysregulation, suggesting that altered CD63 expression may indicate changes in macrophage-mediated vesicle communication during severe inflammation. However, whether CD63 actively contributes to inflammatory polarization or metabolic remodeling remains to be determined. Future studies using macrophage-specific *CD63* loss-of-function models will be required to define its causal role in macrophage activation and lung immunopathology. In addition, although ELAVL1 has been implicated in regulating mRNA stability in immune cells and linked to post-transcriptional control of inflammatory gene expression, emerging evidence suggests that its activity is highly context-dependent and can be rewired by RNA-associated regulatory networks to shape immune cell activation states (Sha et al., 2025; Tanwar et al., 2023). However, its role in coordinating macrophage inflammatory and metabolic programs during viral infection remains incompletely characterized. Our study provides functional evidence that ELAVL1-associated RNA regulatory activity contributes to inflammatory and metabolic remodeling in macrophages. Given that ELAVL1 operates within a complex RNA regulatory network, including m^6^A-associated regulatory mechanisms, its biological effects are likely shaped by cellular context and inflammatory conditions. These findings highlight ELAVL1-associated pathways as potential modulators of pathological macrophage activation, while warranting further investigation into their therapeutic relevance.

This study has several limitations. First, although *JUND* and *ELAVL1* were identified as potential regulators associated with CD63^hi^ macrophage state transitions, their direct downstream targets and regulatory mechanisms remain incompletely defined. Future studies using locus-specific approaches, such as ChIP-seq/CUT&RUN and transcript-specific RNA-binding analyses, will be required to further establish the regulatory network controlling this inflammatory macrophage state. Second, although the LPS-induced acute lung injury model captures key features of macrophage-driven inflammatory amplification, it does not fully reproduce SARS-CoV-2-specific processes, including viral sensing, viral-host interactions, and adaptive immune responses. Importantly, CD63^hi^ MDMs were initially identified from human COVID-19 lung and BALF single-cell transcriptomic datasets, whereas LPS models were used only to evaluate their inflammatory and tissue-damaging potential. Therefore, our findings should not be interpreted as demonstrating that LPS fully models SARS-CoV-2 infection. Future studies using SARS-CoV-2 infection systems, viral RNA mimetics, or patient-derived macrophage models will be required to define virus-specific functions of CD63^hi^ macrophages. Third, although *ELAVL1* perturbation experiments demonstrate its contribution to inflammatory, interferon-associated, and metabolic programs in CD63^hi^ macrophages, the current study lacks macrophage-specific *in vivo* genetic validation of ELAVL1 and CD63. Conditional knockout models combined with rescue experiments will be required to determine the cell-autonomous role of ELAVL1 in macrophage remodeling and to define whether CD63-associated macrophage states represent functional drivers or phenotypic consequences of inflammatory activation. Such studies will also help define the stage-specific roles of this regulatory axis during inflammatory injury and tissue repair. Finally, the therapeutic implications of targeting the ELAVL1-CD63^hi^ macrophage axis remain exploratory. Although modulating macrophage-associated inflammation has shown clinical benefit in severe COVID-19, ELAVL1 represents a complex therapeutic target due to its broad expression and essential roles in RNA regulation, immune homeostasis, and host defense. Systemic inhibition may therefore affect protective immune responses or induce unintended effects. Future studies integrating macrophage-selective delivery strategies, biomarker-guided patient stratification, and rigorous preclinical safety evaluation will be required to determine whether targeting ELAVL1-associated pathways can selectively suppress pathological macrophage activation while preserving immune function.

Collectively, our study identifies CD63^hi^ macrophages as a conserved terminal inflammatory state associated with lung immunopathology in severe COVID-19 and reveals an *ELAVL1*-associated regulatory framework linking post-transcriptional regulation with inflammatory and metabolic macrophage remodeling. These findings refine current models of macrophage plasticity by introducing a fate-commitment perspective in viral inflammation and highlight potential therapeutic strategies for modulating maladaptive macrophage differentiation.

## Data Availability

The datasets presented in this study can be found in online repositories. The names of the repository/repositories and accession number(s) can be found in the article/**Supplementary Material** and **Table 3**. Computational code used for single-cell RNA-seq data analysis.
